# Amyotrophic Lateral Sclerosis: An Emerging Era of Collaborative Gene Discovery

**DOI:** 10.1371/journal.pone.0001254

**Published:** 2007-12-05

**Authors:** Katrina Gwinn, Roderick A. Corriveau, Hiroshi Mitsumoto, Kate Bednarz, Robert H. Brown, Merit Cudkowicz, Paul H. Gordon, John Hardy, Edward J. Kasarskis, Petra Kaufmann, Robert Miller, Eric Sorenson, Rup Tandan, Bryan J. Traynor, Josefina Nash, Alex Sherman, Matthew D. Mailman, James Ostell, Lucie Bruijn, Valerie Cwik, Stephen S. Rich, Andrew Singleton, Larry Refolo, Jaime Andrews, Ran Zhang, Robin Conwit, Margaret A. Keller, for The ALS Research Group

**Affiliations:** 1 National Institute for Neurological Disorders and Stroke, National Institutes of Health, Bethesda, Maryland, United States of America; 2 Coriell Institute for Medical Research, Camden, New Jersey, United States of America; 3 Eleanor and Lou Gehrig MDA/ALS Research Center, Columbia University, New York, New York, United States of America; 4 Massachusetts General Hospital, Charlestown, Massachusetts, United States of America; 5 National Institute on Aging, National Institutes of Health, Bethesda, Maryland, United States of America; 6 University of Kentucky, Lexington, Kentucky, United States of America; 7 California Pacific Medical Center, San Francisco, California, United States of America; 8 Mayo Medical Center, Rochester, Minnesota, United States of America; 9 University of Vermont, Burlington, Vermont, United States of America; 10 National Institute of Mental Health, National Institutes of Health, Bethesda, Maryland, United States of America; 11 National Center for Bioinformatics, National Institutes of Health, Bethesda, Maryland, United States of America; 12 The ALS Association, Calabasas Hills, California, United States of America; 13 Muscular Dystrophy Association, Tucson, Arizona, United States of America; 14 Center for Public Health Genomics, University of Virginia, Charlottesville, Virginia, United States of America; Sanofi-Aventis, United States of America

## Abstract

Amyotrophic lateral sclerosis (ALS) is the most common form of motor neuron disease (MND). It is currently incurable and treatment is largely limited to supportive care. Family history is associated with an increased risk of ALS, and many Mendelian causes have been discovered. However, most forms of the disease are not obviously familial. Recent advances in human genetics have enabled genome-wide analyses of single nucleotide polymorphisms (SNPs) that make it possible to study complex genetic contributions to human disease. Genome-wide SNP analyses require a large sample size and thus depend upon collaborative efforts to collect and manage the biological samples and corresponding data. Public availability of biological samples (such as DNA), phenotypic and genotypic data further enhances research endeavors. Here we discuss a large collaboration among academic investigators, government, and non-government organizations which has created a public repository of human DNA, immortalized cell lines, and clinical data to further gene discovery in ALS. This resource currently maintains samples and associated phenotypic data from 2332 MND subjects and 4692 controls. This resource should facilitate genetic discoveries which we anticipate will ultimately provide a better understanding of the biological mechanisms of neurodegeneration in ALS.

## Introduction

Amyotrophic lateral sclerosis (ALS) is an adult-onset neurodegenerative disease that causes paralysis. Presently, it is incurable and rapidly progressive, with a mean survival of 4–5 years from onset [1], [2]. Approximately 10% of ALS cases are inherited in a Mendelian fashion (Table 1), with the remainder of cases apparently sporadic [3]–[13]. Generally, familial forms are clinically and pathologically indistinguishable from sporadic forms [14].

**Table 1:** Loci Linked to Mendelian Causes of ALS.

Gene name	Locus Name	Inheritance Mode	Locus	Selected References
Cu/Zn superoxide dismutase (SOD1)	ALS1	AD	21q22.1	43
ALSIN	ALS2	AR	2q33.1	40
Senataxin (SETX)	ALS4	AD	9q34.13	3
Dyncatin (DCTN1)		AD	2p13	4,5
Angiogenin (ANG)			14q11.1-q1.2	6
Microtubule associated protein tau (MAPT)*		AD	17q21.1	45
	ALS3	AD	18q21	14,41
	ALS5	AD	15q15.1-q21.1	46
	ALS6	AD	16q21	10
	ALS7	AD	20p13	10
Vesicle associated protein B (VAPB)	ALS8	AD	20q13.33	11
	ALS-FTD	AD	9q21-22	12
	ALS-FTD	AD	9p13.2-21.3	13

* Multi-system neurodegeneration dominated by fronto-temporal dementia.

AD = Autosomal Dominant. AR = Autosomal recessive.

It is suspected that the sporadic forms of neurodegenerative disorders are caused by multiple genetic variants that individually make relatively weak contributions to risk. Genome-wide association scans have been proposed as a way to assess a large number of cases and controls to discover genetics factors with weak contributions to risk for disease. Recent advances in single nucleotide polymorphism (SNP) genotyping and haplotype mapping have made -genome-wide association (GWA) scans feasible. The distribution of familial versus sporadic disease in ALS is similar to other adult-onset neurodegenerative diseases such as Parkinson's disease and Age-related Macular Degeneration, in which whole genome approaches have been successful [15]–[17]. Towards the goal of allowing whole genome technology to be applied in gene discovery in ALS, we have developed a publicly accessible national resource of biological samples linked to individual phenotypic datasets; a subset of this collection has already been associated with publicly posted genotypic data (via the DbGaP database of genotype and phenotype, http://www.ncbi.nlm.nih.gov/entrez/query.fcgidbgap) [18].

Because ALS is predominantly sporadic, environmental triggers may be involved in disease initiation. With a few exceptions suggested in the literature, such as participation in professional sports [19] and perhaps a history of smoking, no clear environmental risk factors have been identified for ALS, perhaps because the triggers may act only in genetically susceptible individuals. GWA studies to identify susceptibility are also useful in evaluating the interaction between genetic risk factors and putative environmental triggers, as long as adequate data on environmental exposures and lifestyle are also collected. Apart from information concerning smoking history, the current dataset collected on each patient does not include detailed risk factor information. However, there is scope for the dataset to be expanded in the future to include these putative triggers.

## Methods

### Description of the Collaboration

Although ALS is the most common MND, it is still a relatively rare disease with an incidence of around 1.6 per 100,000 in the United States [20]. Thus, collaboration is essential to obtain a sufficient sample size to allow statistically meaningful genetic studies. NINDS has created a DNA and cell line repository towards the goal of creating large sample size collections to allow the study of complex disease genetics. In 2004, ALS was added as one of the diseases banked (along with Parkinson, Stroke, and Epilepsy, and most recently, added in 2007, Tourettes). To facilitate complex gene discovery in ALS, the NINDS Repository (under a contract with Coriell Cell Repositories), in collaboration with The ALS Association (ALSA), The Muscular Dystrophy Association (MDA) and academic scientists from 62 centers across the United States, developed a program for evaluation of individuals affected with ALS, neurologically normal controls, and banking of their blood samples and clinical data. This unique public-private collaboration facilitated leverage of resources to develop this collection broadly across the United States. Germane to the success of the venture was the high level of interaction and communication among the clinical groups regarding subject evaluation, sample collection, and other aspects of the project. This allowed standardization to be maximized and also maintained enthusiasm at the sites for a “living” project. This model is likely to be useful for other researchers who are interested in to collecting, banking and sharing DNA for gene discovery in rare disorders as well as those considering banking other types of biological material or data.

Many of the clinicians in this collaboration are members of the ALS Research Group (ALSRG) [20]. The ALSRG is a group of investigators whose stated mission is to advance basic and clinical research of ALS. When NINDS invited applications for an administrative supplement to ongoing National Institutes of Health (NIH)-funded studies to support sample collection towards gene discovery in neurological disorders (NOT-NS-03-016), the ALSRG recognized the opportunity for a collaborative response [20]. The contributions of the ALSRG have ‘added value’, because the samples and data they submit are made available immediately to the broader scientific community [21]. Investigators who had been collecting and banking samples as a part of the overall NINDS -funded effort in ALS gene discovery prior to the NOT-NS-03-016 initiative also have contributed to this effort (JH,AS,BT).

## Results

Together, we have built a collection of DNA and cell line samples from well-characterized cases and controls; this collection is made publicly available to researchers while protecting the privacy of all participants. Biomaterials from 2332 individuals with amyotrophic lateral sclerosis (49% men, 51% women), as well as samples from 4692 neurologically normal control subjects are currently in the collection as of September, 2007 (Additional data on subject characteristics can be found in Appendix S1 D). Of note, all of the samples and data collected in the ALSRG project were done under an agreement for immediate release for research to all bona fide researchers. Phenotypic data corresponding to samples are also posted in the NINDS repository web-based catalogue (http://ccr.coriell.org/Sections/Collections/NINDS/SsId10). Additional samples and data will be released in the next several months as they are processed for DNA and data cleaning. Samples may be purchased individually or as pre-compiled panels/plates, each consisting of 92 subjects. There are now six such panels of individuals diagnosed with ALS (plate catalog numbers NDPT025, NDPT026, NDPT027, NDPT028, NDPT029 and NDPT030) and nine panels of neurologically normal control subjects (plate catalog numbers NDPT002, NDPT009, NDPT019, NDPT020, NDPT021, NDPT022, NDPT023, NDPT024 and NDPT031).

Peripheral blood samples were collected and all are processed to produce EBV-transformed lymphoblastoid cell lines. The ability to derive cell lines from banked blood specimens allows for broad sharing as well as for gene expression studies in order to determine the biological relevance of genetic discoveries and other follow-up studies. Additionally, the availability of cell lines ensures a perpetual source of DNA, which allows broad sharing to occur across the scientific community.

It is expected that all genotypic data generated from analysis of samples in this collection will also be made available through dbGaP (http://www.ncbi.nlm.nih.gov/entrez/query.fcgidbgap). Already, a first stage analysis using genome wide SNP technology has been carried out using 276 samples of sporadic ALS cases and 271 neurologically normal controls from this collection effort [18]. Briefly 555 352 unique SNPs were assayed in each DNA sample using the Illumina Infinium II HumanHap550 SNP chip. More than 300 million genotypes were produced in 547 participants and this genotyping data is in the process of being posted to dbGaP, the NCBI hosted public database for genotyping and phenotyping (as described in more detail below). It will be linked from DbGaP to the NINDS Repository samples and vice versa. While no single locus was identified as associated with an increased risk of disease in that first stage analysis [18], these genotyping data will add greatly to the value of this resource for additional hypothesis generation.

## Discussion

### Sufficient Sample Size to Address Complex Genetic Risk

Recent technological advances in genomics coincide with increasing recognition of the importance of very large cohorts for studying complex genetic effects [22]. How many subjects are needed for disease gene discovery in ALS? The answer to that question depends on several factors, including genetic architecture (number of genes, their effect size, and interactions with other genes and environmental risk factors), potential disease heterogeneity (genetic and environmental), and proposed study design. It has been estimated that, for a statistical power of 80%, GWA requires ∼3,000 samples and ∼3,000 controls to discover alleles with frequencies of >0.2 or <0.8 that associate with disease at an odds ratio of >1.3 [22]. Similarly, analyzing specimens from a minimum of 2,000 cases and 2,000 controls should allow identification of alleles with approximately a 1.5-fold or greater relative risk. Currently, the NINDS Repository has achieved a collection size consistent with this latter more conservative estimate. To design a study capable of detecting gene-gene or gene-environment interactions, the sample size required to maintain power would be increased by at least 4-fold. Moreover, replication of experimental findings depends upon the availability of populations independent of the original cohort. Thus, there are clear reasons for large collections of specimens, and for expanding the NINDS Repository ALS collection in the future should that be possible.

It should be emphasized, however, that even with recent advances in genomics and bioinformatics, the number of subjects needed cannot be precisely predicted.

This is because we do not know the number of loci involved in ALS, whether rare or common alleles are more prevalent in terms of conferring susceptibility, nor their allele frequency and penetrance. This complexity is illustrated by the role of SOD1 in ALS susceptibility. There are SOD1 alleles that are inherited in a Mendelian fashion with ALS [2], [43]. These represent a small minority of ALS cases [2]; however, there are SOD1 alleles that confer susceptibility with incomplete penetrance [24]–[26]. Thus, a single gene can contribute to “Mendelian” and “complex” genetic causes of ALS. It is not surprising, therefore, that many diseases, including ALS and other neurodegenerative disorders, have multiple similar but not identical clinical profiles that may reflect differences in underlying genetic causes. Finally, different genetic causes may be distributed among sub-populations or strata defined by gender, age of onset, site of onset, race or ethnicity, or even, importantly, therapeutic responsiveness and survival. These strata are potentially valuable classification variables, and it is likely that the more stratified a population of subjects, the larger the sample size needed for study [22].

### Phenotyping

It is possible that sporadic ALS represents a number of biological entities, with overlapping clinical features [27]. This, together with the requirement to enroll thousands of subjects, means that clearly defined phenotypic definitions, standardized data collection, and rigorous data management are essential to a collaborative effort such as this one [27]. Detailed phenotypic data will be germane to further analyses as well, as there are likely endo-phenotypes not yet known to be biologically important but which are critical for understanding complex disease. Furthermore, excellent clinical assessment in the field maximizes information gained from these collections, and avoids “wasted efforts” which can occur in underpowered studies [28]. Fortunately, in the ALS academic community, clinical trials networks have facilitated the use and application of standardized clinical criteria, such that collection of large, well-characterized populations in ALS for gene discovery is achievable.

While ALS is the most common form of motor neuron disease, other less common motor neuron and systemic diseases can confound a diagnosis of ALS. Current diagnostic criteria for ALS are based on clinical assessments, and require the presence of both upper motor neuron (UMN-spasticity, hyper-reflexia, Babinski) and lower motor neuron (LMN muscle atrophy, fasciculations, weakness) involvement [27], [29]. The level of diagnostic certainty rests on the extent of UMN and LMN signs. These signs, their severity, and associated findings form the basis of the World Federation of Neurology (WFN) El Escorial diagnostic criteria [27], [30]. Per these criteria, “Definite ALS” is based on the coexistence of UMN signs and LMN signs in the bulbar and spinal regions. These criteria may not be fulfilled on an initial visit to the clinic and thus longitudinal follow-up may be needed to reach an accurate clinical diagnosis [31]. Such follow-up is possible in the cohort collected here because of the data-basing capabilities of the NINDS Repository as well as the patient management of the ALSRG, and is ongoing for this collection. Additionally, since clinical collection sites for the ALS collection are primarily led by specialists in ALS, the specimens included in the NINDS DNA and Cell Repository have highly accurate clinical data.

For subject inclusion, complete NINDS Repository Clinical Data Elements (CDEs) are required. These elements were developed to permit researchers using the specimens to apply the El Escorial Criteria for the diagnosis of ALS at more than one level of stringency in a standardized fashion [26], [27], [29]. Additionally, these were designed towards allowing broad pooling of multiple sample sets, since there are many international groups collecting samples for ALS gene discovery with which those from this effort could ultimately be pooled to achieve larger sample sizes and thus greater power to detect genes of risk. These CDEs also query exclusionary features, such as electrophysiological, CSF, imaging or other findings suggestive of confounding diagnoses (Appendix S1, B). Data dictionaries have been designed by the collaboration (EK, KB) and are publicly available to allow rapid referencing of all phenotypic terms (http://ccr.coriell.org/Sections/Collections/NINDS/CDE/mnd_dd.aspxPgId347). This further enhances the value of the biological specimens, guides future submissions, and facilitates phenotype-genotype correlations and sharing across collections.

In addition to precise and detailed phenotyping of affected individuals, well-designed collections of unaffected (control) subjects are crucial to genome-wide association studies of ALS and other disorders. The phenotypic data collected on individual controls in this collection is designed for use in gene discovery efforts in ALS as well as other neurodegenerative disorders. CDEs for unaffected individuals likewise were designed towards standardization, providing some neuro-psychiatric, medical and family history assessments (Appendix S1, C). For example, all subjects, whether case or control, are queried regarding family history of ALS, Parkinsonism, dementia, Alzheimer's disease, and other neurological disorders. Identifying and enrolling large numbers of control subjects in these studies benefits from having a large collaborative team effort. This is valuable since “apparently healthy”, “neurologically normal” individuals, who would be suitable for use as control subjects, are not routinely seen in an academic neurology practice. This approach to using controls for multiple studies has recently been shown to be valuable for gene discovery. Using a shared set of ∼3,000 controls, case-control comparisons were used to successfully identify independent significant association signals in bipolar disorder, coronary artery disease, Crohn's disease, rheumatoid arthritis, type 1 diabetes, and type 2 diabetes [32]. That study supports our strategy of building a carefully assessed shared control group represents a scientifically sound and highly effective approach to GWA analyses of multiple disease phenotypes.

There are some caveats resulting from this approach to control collection. First, restrictions of enrollment (i.e., absence of a medical or first degree family history of neuro-psychiatric disorder) can slow recruitment. Second, it is not simple to classify a subject as “free of neurological disease” since most control subjects are evaluated only once, and neurological disease symptoms may arise late in life. Finally, and perhaps most importantly, ALS patients were recruited regardless of whether there was a family history of neurological disease, while controls were not included if they had a family history of neurological disease. This restriction was put in place in order to facilitate broad sharing of control samples across neurological disease entities. However, for optimal WGA studies it can be argued that it is important to include only ALS probands that do not have a family history of another neurological diagnosis. To address this concern DNA panels being designed by the Repository for WGA ALS studies for high throughput screening from this collection segregate ALS cases with a family history of neurological disease from those with no family history.

### Consent and Patient Protection

Patient consent and privacy in genetic studies is an evolving field of science policy. When the collaboration was established, care was taken to assure compliance with existing regulations, while planning for the potential of broad usage. In all cases the collection, storage, distribution and use of human specimens and data were conducted in accordance with all applicable regulations including: 45 CFR Part 46: the FDA human subjects regulations 21 CFR Parts 50, 56, and 812: the Health Insurance Portability and Accountability Act (HIPAA) Privacy and Security Rules (45 CFR Parts 160 and 164): and any state and local laws. Under 45 CFR Part 46, research use of specimens and data that are not identifiable, and for which there are no links to individually identifying information, is not considered to be human subject research. Because individual identifiers are not accepted into the public database, the data being shared are not considered human subject data. Additionally, some repositories, including the NINDS Repository, are operated under contracts to non-billable entities, i.e., entities that do not provide clinical care. In those cases, the Health Insurance Portability and Accountability Act (HIPAA) does not apply. Nonetheless, this project and all others at the NINDS Repository maintain HIPAA compliance towards the goal of consistent, stringent respect for individual privacy (see http://privacyruleandresearch.nih.gov/research_repositories.asp and OHRP: Guidance on Research Involving Coded Private Information or Biological Specimens, issued August 10, 2004 http://www.hhs.gov/ohrp/humansubjects/guidance/cdebiol.pdf).

In following the requirements for human subject research, subject ascertainment and sample and data collection prior to repository submission requires informed consent and oversight by an Institutional Review Board (IRB). Obtaining informed consent is a crucial element for assuring that individuals are aware of the relative risks and benefits of the research and that they are free to choose or refuse to participate [33]. The risks to participants include potential breach of privacy and confidentiality. While the data are systematically de-identified in terms of traditional identifiers, genotyping could theoretically be used to identify an individual were that person to provide a second sample to a third party for comparison. There are also minor physical risks related to blood drawing, such as bruising and discomfort at the site of phlebotomy. While it is highly unlikely that any individual will suffer from a psychosocial standpoint as a result of participating, there is the putative risk that if individuals with ALS as a whole are identified as having a particular gene variant, some might experience stress in this regard.

The relative benefits to society are considered explicitly in discussions with subjects. In the informed consent process for this study, it is stressed that in a period of months to years this work is unlikely to lead to direct benefit for any individual human subject, but over many years to decades it may improve our understanding ALS and ultimately result in health benefits for ALS patients. Counter to negative speculation regarding anxiety and resistance to participate in genetics studies, we anecdotally found that subjects were extremely enthusiastic to participate. In fact, patients and family members of those with ALS continue to request participation (KAG). However, such requests cannot typically be met, because this particular project was funded under an NINDS initiative (NOT 03-016) which has expired, and so, the ALSRG is not currently banking ALS samples in an ongoing fashion. Nonetheless, NINDS continues to fund gene discovery projects in ALS, and those other projects which are investigator initiated (R01) funded projects continue to bank ALS samples as part of those studies. It is hoped that additional large scale, ongoing sample collections can be resumed, once it is clear that even larger sample sizes are necessary and will be further used in gene discovery.

All samples were collected using consent forms approved by local IRBs. The NINDS repository has developed a sample consent form (http://ccr.coriell.org/Sections/Support/NINDS/icmodel.aspxPgId317) based on the parameters suggested by Beskow et al [33] and updated based on the discussions at a recent NIH workshop, “Multi-Institute Symposium on the Application of Genomic Technologies to Population-Based Studies” (June 2006 internal NIH meeting, KG personal communication). This template offers a useful starting point for investigators developing consent forms for genetic studies in ALS and many other disorders. Additionally, the NINDS Repository suggests specific elements for consent forms to assure that key points are raised (Appendix S1, E). Stemming from that, and because this collection was established in collaboration with a clinical trials network (ALSRG), an IRB protocol and template was developed by the ALSRG that allowed relatively uniform and coordinated IRB approval processes for all participating sites [21].

Often, longitudinal follow-up of individuals suspected of having ALS is needed to reach an accurate diagnosis. However, patient protections make collection of longitudinal data difficult for a centralized repository; as such follow-up data must be collected without breaking subject anonymity. Therefore, longitudinal data collection depend on the voluntary submission of such data by contributing investigators, which was approved by most local IRBs and is described in these consent forms.

### Processing of samples submitted to the NINDS Repository

Limited access to biomaterials collected by individual laboratories and projects has presented a major roadblock in the past to genome-wide analyses of complex diseases, including gene discovery in sporadic ALS. The NIH and other contracting agencies have addressed the need for such biomaterials by funding non-profit repositories to receive, manage and distribute human biomaterials, including the NINDS Repository. Over the last 30 years, the Coriell Institute has played a leading role in establishing quality control guidelines for Cell and DNA DNA repositories.

A key process in the Coriell repositories relies on an approach that was developed over 30 years ago, in which EBV infects and transforms B lymphocytes present in whole blood [33]. The transformed lymphoblasts from each individual subject represent a renewable source of genetic material. Both immortalized cell lines and the DNA derived from them are a valuable resource for the biomedical research community at large. Additionally, in some cases, availability of cell lines with associated genotypic and phenotypic information represents a second-generation resource for mRNA- and protein-expression analyses and other cell-based studies aimed at follow-up of genetic “hits”.

The Coriell Institute has established a set of quality control procedures to ensure that each sample is processed in an identical manner and with the same high standards.

Coriell Cell Repositories have established a set of quality control procedures to ensure that each sample is processed in an identical manner and with the same high standards. For the NINDS Repository, two tubes containing blood samples are submitted per subject. Each blood sample is assayed for length polymorphism at 6 independent short tandem (STR) repeat loci. As a first level of control, the STR profiles from the two blood tubes must be identical to each other. Moreover, all derived biospecimens, i.e. DNA and cell culture, must also match the STR profile from the original blood. Gender is determined by a PCR assay, and is compared with declared gender. Finally, Coriell uses STR profiles, gender, and year of birth to establish singularity for each submission, thus avoiding banking the same subject twice under different catalog numbers. Care is taken to ensure that identical twins are not eliminated by this process.

### Advances in Genomic Medicine

Technical advances in molecular biology over the past 20 years, including the advent of polymerase chain reaction (PCR), discovery of SNPs, and automation have provided essential tools for high-throughput genome-wide studies. Population-based maps of the correlations among SNPs (linkage disequilibrium) are being developed in an ongoing fashion [34]. The human genome is thought to contain at least 10 million SNPs, about one in every 300 bases. Theoretically, researchers could hunt for genes using a map listing all 10 million SNPs, but there are major practical drawbacks to that approach, including expense and data management. Fortunately, the HapMap project has accelerated disease related gene discovery as well as many other projects [35]. HapMap has identified blocks of cis-linked SNPS that, in a given ethnic population, generally segregate as a group. This allows researchers to use a few ‘tag SNPs’ to identify a unique block of the genome (a haplotype block). As a result, rather than needing to sequence all 10 million SNPs in the human genome, only 300,000 to 600,000 tag SNPs are needed to efficiently identify the haplotypes in the human genome [36]. Already, this approach is finding widespread use in fine mapping of genetic disorders, in the delineation of genetic influences in multifactorial diseases such as breast cancer, myocardial infarction, type 2 diabetes, and asthma, and as genetic markers to predict responses to drugs and adverse drug reactions [37].

Performing whole genome association (WGA) scans in ALS, as well as in other disorders, depends on robust technologies for analysis of individual SNP variants. Several SNP genotyping platforms exist, and the use of more than one in gene discovery should have the added benefit of comparing these alternative platforms.

### Bioinformatics

Broad access to data and biomaterials is one of the key principles in realizing the potential of genomic science [38]. Underpinning uniform public access are bioinformatics solutions for managing the phenotypic and genotypic data. The NINDS Repository was the first bio-repository to make disease-related, genome-wide genotyping data completely and publicly accessible (in Parkinson's disease and Control subjects, see references [16], [38]). This genotype/phenotype dataset (https://queue.coriell.org/Q/snp_index.asp), initially posted in March 2006, and now also available via dbGaP (http://www.ncbi.nlm.nih.gov/entrez/query.fcgidbgap) has generated considerable interest in the scientific community, and has already been accessed by about a thousand different researchers across the globe. This underscores the value of public availability of this data.

Such bioinformatics solutions are dependent on the existence of a scalable and extensible informatics infrastructure. The information management system should meet the requirements of real-time data capture, collection site management, chain of custody handling, and operational efficiency for a large number of samples, each of which is linked to individual data. Moreover, as an integrated solution, the system must manage the genotypic and phenotypic data associated with bio-specimens under compliance with all relevant privacy laws. The information systems design must not only consider the quality but also the accessibility of the bio-specimens and associated data. The system also needs to have the ability to integrate with other databases as both a source and a recipient of data. The NINDS Repository bioinformatics system meets all of these requirements as does the National Center for Biotechnology Information (NCBI)'s dbGaP project.

DbGaP meets all of these requirements on a broader scale by providing analysis and retrieval resources for many types of data, including genetic and other biological data. Furthermore, it provides a standardized approach for public sharing of anonymized genotype and phenotype data across NIH. This broadly available, standardized, and scalable resource prevents redundancies and allows uniform approaches to privacy and access [40], [41]. The ALS study phenotypic data, data dictionary, and supporting documents are soon to be posted on the dbGaP site (http://www.ncbi.nlm.nih.gov/entrez/query.fcgidbgap). DbGaP has two orders of access (open, and controlled) which permit broad release of non-sensitive data in the first case, but also providing oversight and investigator accountability for sensitive data sets involving personal health information in the second. Summaries of the ALS and control subject CDEs, the ALS data dictionary, and other documents used for this collection and analyses will be made available to investigators via dbGaP. It is expected that investigators who use the bio-specimens banked at the NINDS repository for future genotyping will submit their genotyping data to dbGaP once it is available. The dbGaP database links to the database at the NINDS that also has searchable phenotypic data and associated biological materials which would allow sub-set analysis and biological expression study follow-up of ‘hits’.

### Summary

ALS research has entered an era of discovering complex genetic causes of disease. Many genes in familial ALS have been identified or mapped. However, most cases of ALS are sporadic, and genetic factors may contribute to the risk for disease. WGA studies are feasible given the advances in SNP and other genotyping technologies. However, to carry WGA forward with adequate power to detect true effects, a very large sample size is needed–probably thousands of unique affected subjects and thousands of controls. It is clear that bio-repositories play an important role in this effort, as do bioinformatics resources, clinical consortia, and a willingness to share data broadly. NINDS has succeeded in creating a resource containing thousands of unique DNA and immortalized cell line samples from individuals with ALS and corresponding control subjects. The strong tradition of clinical collaboration in ALS set the foundation for building this biological sample bank and phenotypic dataset which now allows whole genome studies in ALS to occur. This effort was based upon a collaboration comprised of academic investigators, NIH staff, clinicians, and non-government organizations to create an infrastructure by which biomaterials and associated phenotypic and genotypic data which have been collected can be distributed responsibly with minimal barriers to researchers. Bioinformatics development at the NINDS Repository has allowed organization of searchable phenotypic data and sample sets, and integrates with other database projects, such as NCBI's DbGaP. A first stage WGAS analysis has been undertaken with a subset of these samples, which will facilitate further hypothesis generation and genetic study of this and other sample collections.

As with all genome-wide approaches to complex disease, there are continued challenges regarding determination of optimal sample sizes for affected and control populations. There is no single paradigm for gene discovery and no single ideal study design or analytical approach. Additional sample sets may be needed for validation of initial studies both in the same populations as well as in other ethnic groups. We anticipate that the identification of disease-specific genes will provide opportunities to develop early diagnostic measures, suggest surrogate markers of disease progression, and supply targets for therapeutic discovery. In summary, the NINDS Repository in collaboration with dbGaP allows genotype, phenotype, and biological specimens to be associated and distributed as a public resource. Future studies will likely explore gene-gene and gene environment interactions. The inclusion of pharmacological response or significant drug exposure in future clinical datasets of patients enrolled in genetic studies with further enrich this collection. Our experience can act as a springboard for such future endeavors.

## Supporting Information

Appendix S1 (0.38 MB DOC)
