# Supplementary material for: Amyotrophic Lateral Sclerosis: An Emerging Era of Collaborative Gene Discovery
Source: PLoS One. 2007 Dec 5;2(12):e1254. doi: 10.1371/journal.pone.0001254 (PMC2100166; doi:10.1371/journal.pone.0001254)
Supplement: Appendix S1 — (0.38 MB DOC) [file pone.0001254.s001.doc]

Appendix S1

### A. Additional ALS Research Group Members (in order of number of submissions):

Catherine Lomen-Hoerth, University of California San Francisco; Zachary Simmons, Pennsylvania State-Hershey Medical Center; Daniel S. Newman, Henry Ford Hospital; Richard J. Barohn, University of Kansas; Brian Crum and J. Clarke Stevens, Mayo Clinic Rochester; Ericka P. Simpson, Methodist Neurological Institute; Kevin B. Boylan, Mayo Clinic Jacksonville; Leo McCluskey, University of Pennsylvania; Richard S. Bedlack, Duke University Medical Center; E Peter Bosch, Mayo Clinic Scottsdale; Paul E. Barkhaus, Medical College of Wisconsin; Allitia Dibernardo, Massachusetts General Hospital; James B. Caress, Wake Forest University Baptist Medical Center; David Lacomis, University of Pittsburgh Medical Center; Alan Pestronk, Washington University; Jeremy M. Shefner, State University of New York; Nicholas J. Maragakis, Johns Hopkins University; Daragh Heitzman, Texas Neurology, Physicians Associates; Kimberly L. Goslin, Providence Medical Center; Carlayne E. Jackson, University of Texas San Antonio; Jonathan D. Glass, Emory University; Tahseen Mozaffar, University of California Irvine; Tulio E. Bertorini, University of Tennessee; David A. Chad, University of Massachusetts; Jaya R. Trivedi, University of Texas Southwestern; Kourosh Rezania, University of Chicago; Terry D. Heiman-Patterson, Drexel University; Laurie Gutmann, West Virginia University; Jeffery Rosenfeld, Carolinas Medical Center; Benjamin R. Brooks, University of Wisconsin; Ghazala Hayat, Saint Louis University; John E. Chapin, University of New Mexico; Stacy A. Rudnicki, University of Arkansas; Yadollah Harati, Baylor College of Medicine; Sandeep S. Rana, Allegheny General Hospital; Ashok Verma, University of Miami; James A. Russell, Lahey Clinic; Erik P. Pioro, Cleveland Clinic; Charles A. Thornton, University of Rochester Medical Center; Laura Sams, University of Cincinnati; John Kelly and Elham Bayat, George Washington University; Praful M. Kelkar and Ezzatollah T. Shivapour, University of Iowa; Stephen N. Scelsa, Beth Israel Medical Center; David Walk, University of Minnesota; Amanda C. Peltier, Vanderbilt University; George Sachs, Rhode Island Hospital; Jerry M. Belsh, Robert Wood Johnson Medical Center; Michael C. Graves, University of California Las Angeles; Nimish J. Thakore, Metro Health Medical Center; Harris T. Brent, Dartmouth Hitchcock Medical Center; Charles Cho, Stanford University; James P. Wymer, Saint Peter’s Hospital; Jau-Shin Lou, Oregon Health Sciences University; Michael D. Weiss and Gregory S. Carter, University of Washington; Carmel Armon, Baystate Medical Center; Thomas R. Vidic, Elkhart Clinic; Mark B. Bromberg, University of Utah; Dale J. Lange, Mount Sinai Hospital and Medical Center.

**B. Clinical Data Elements NINDS Repository for Affected Individuals**

**C. Clinical Data Elements, NINDS Repository, Control Subjects**

D. Geographical Distribution of Cases and Controls

| **Geographical Distribution** | **ALS Cases** | **Controls (All)** |
| --- | --- | --- |
| Location 1: New England | 154 | 242 |
| Location 2: Middle Atlantic | 387 | 383 |
| Location 3: East North Central | 228 | 250 |
| Location 4: West North Central | 175 | 191 |
| Location 5: South Atlantic | 279 | 410 |
| Location 6: East South Central | 100 | 112 |
| Location 7: West South Central | 198 | 120 |
| Location 8: Mountain | 63 | 72 |
| Location 9: Pacific | 245 | 290 |
| no value* | 104 | 2233 |
| **Sociodemographic** | **ALS Cases** | **Controls (All)** |
| **Age (yrs, mean +/- std)** | 57 +/- 12 | 51 +/- 15 |
| **Gender (% male)** | 49 | 47 |
| **Race (%)** |  |  |
| African American | 4.0 | 8.6 |
| American Indian | 0.4 | 0.3 |
| Asian | 9.6 | 9.4 |
| Caucasian | 82.5 | 78.9 |
| no value | 0.4 | 0.3 |
| other | 2.2 | 1.4 |
| pacific islander | 0.1 | 0.2 |
| unknown | 0.8 | 0.6 |
| **Hispanicity (%)** |  |  |
| Hispanic/Latino | 3.7 | 3.1 |
| Not Hispanic/Latino | 94.6 | 92.3 |
| No Value | 1.7 | 4.5 |
| **Confirmed Mutations (#)** |  |  |
| SOD1 | 15 | N/A |
| other | 2 | N/A |
| **Positive Family History (%)** |  |  |
| absent | 86.1 | 95.8 |
| unknown | 1.9 | 2.6 |
| present | 9.3 | 1.5 |
| present 1st degree relatives | 4.8 | 0 |

*In some cases geographical information cannot be collected due to HIPPA and/or local IRB regulations.

Regions are as defined by the U.S. Census Bureau (<http://www.census.gov/geo/www/us_regdiv.pdf>). Data are from the NINDS Repository as of 6/4/07.

Data are from the NINDS Repository as of 6/4/07.

**E. Sample Consent Form Elements for Gene Discovery Projects**

- The blood sample will be submitted to the Coriell Cell Repositories, a research resource supported by the National Institutes of Health/ National Institute of Neurological Disorders and Stroke
- The blood will be used for the preparation of an immortalized cell line from which DNA will be prepared.
- The cell line and DNA and accompanying data including genotyping data will be distributed to scientists including those in research, teaching, and industry.
- The sample will be kept indefinitely.
- The sample and the accompanying information, including the clinical and genetic information, will be used for study of many disorders, not just the one that the subject may have.
- The clinical data and the genetic data will be housed anonymously in a public database.
- The identities of subjects will not be stored and that the samples will be de-identified, (in accordance with HIPAA regulations).
- There is a risk that someone could use information from the sample you submitted, via DNA, to identify you if it were matched with another DNA sample provided by you. However, any user of this sample must agree not to use it for that purpose, and the risk, while real, is small.
- You have the right to withdraw from this research project at any time. If possible, any samples you have contributed will be discarded if you request this; however, because of the sample masking, we may not always be able to identify which samples were donated by you. Your withdrawal from the study will in no way affect access to medical care for which you are otherwise eligible.
